# Cost-effectiveness of the timing of respiratory extracorporeal membrane oxygenation initiation in pediatric patients

**DOI:** 10.1590/1984-0462/2026/44/2025275

**Published:** 2026-08-24

**Authors:** Gianmarco Camelo-Pardo, Alejandra Mendoza-Monsalve, Maricel Licht-Ardila, Angélica Lucero Ortiz-Córdoba, José Rafael Almarales-Navarro, Alexandra Hurtado-Ortiz, Anderson Bermon, Edgar Fabián Marique-Hernández

**Affiliations:** a Fundación Universitaria, Fundación Cardiovascular de Colombia, Piedecuesta, Santander, Colombia.; b Fundación Universitaria del Área Andina, Bogotá, Colombia.

**Keywords:** Extracorporeal membrane oxygenation, Mortality, Respiratory distress syndrome, Intensive care units., Oxigenação por membrana extracorpórea, Mortalidade, Síndrome de desconforto respiratório, Unidades de terapia intensiva.

## Abstract

**Objective:**

The aim of this study was to assess the cost-effectiveness of early versus late initiation of extracorporeal membrane oxygenation (ECMO) in pediatric patients with acute respiratory distress syndrome admitted to the intensive care unit.

**Methods:**

A cost-effectiveness analysis was conducted from the hospital provider perspective, comparing early (≤7 days) versus late (>7 days) initiation of ECMO. Direct medical costs and 90-day survival were incorporated into a decision tree to estimate the incremental cost-effectiveness ratio. Deterministic and probabilistic sensitivity analyses were performed using a willingness-to-pay threshold equivalent to three times the Colombian gross domestic product per capita.

**Results:**

Among 78 patients, most patients received early ECMO initiation, and this strategy was associated with similar mortality and comparable total direct medical costs compared with late initiation. Probabilistic simulations indicated a higher probability of cost-effectiveness for early initiation across different willingness-to-pay thresholds. Cost parameters were the main drivers of variability in the incremental cost-effectiveness ratio.

**Conclusions:**

No statistically significant differences were observed between early and late ECMO initiation in survival or costs. A possible economic trend favoring early initiation should be interpreted cautiously, given limited statistical power.

## RESUMO

**Objetivo:**

Avaliar a custo-efetividade da oxigenação por membrana extracorpórea (ECMO) de iniciação precoce *versus* tardia em pacientes pediátricos com síndrome do desconforto respiratório agudo internados em unidade de terapia intensiva.

**Métodos:**

Realizou-se uma análise de custo-efetividade sob a perspectiva do prestador hospitalar, comparando a iniciação precoce (≤7 dias) com a tardia (>7 dias) da ECMO. Os custos médicos diretos e a sobrevivência em 90 dias foram incorporados a uma árvore de decisão para estimar a razão incremental de custo-efetividade. Análises de sensibilidade determinísticas e probabilísticas foram realizadas utilizando um limiar de disposição a pagar equivalente a três vezes o produto interno bruto *per capita* da Colômbia.

**Resultados:**

Entre 78 pacientes, a iniciação precoce representou a maioria dos casos e apresentou mortalidade semelhante e custos diretos totais comparáveis à iniciação tardia. As simulações probabilísticas indicaram maior probabilidade de custo-efetividade para a iniciação precoce em diferentes limiares de disposição a pagar. Os parâmetros de custo foram os principais determinantes da variabilidade da razão incremental de custo-efetividade.

**Conclusões:**

Não foram observadas diferenças estatisticamente significativas entre a iniciação precoce e tardia da ECMO em termos de sobrevivência ou custos. Uma possível tendência econômica favorável à iniciação precoce deve ser interpretada com cautela em razão do poder estatístico limitado.

## INTRODUCTION

Pediatric Acute Respiratory Distress Syndrome (PARDS) is an acute and diffuse inflammatory injury that leads to increased pulmonary vascular permeability, increased lung weight, and loss of aerated lung tissue.^1,2^ This process damages the alveolar and endothelial epithelium, increasing the permeability of the alveolocapillary membrane and resulting in pulmonary edema, which leads to severe hypoxemia and acute respiratory failure.^2,3^ PARDS is a significant cause of morbidity and mortality in the pediatric population,^4^ with incidences ranging from 2 to 12.8 cases per 100,000 people per year and mortality rates that can vary between 18 and 27%.^4,5^

The syndrome is associated with multiple conditions, including pneumonia, sepsis, trauma, burns, pancreatitis, toxic inhalation, and transfusion-related reactions, although viral respiratory infections remain the most frequent etiology.^6,7^ The diagnosis and management of PARDS require a multidisciplinary approach.^8,9^ Standard therapeutic strategies include treatment of the underlying condition, invasive mechanical ventilation, and prone positioning, with neuromuscular blockade reserved for severe cases.^10^ Despite these measures, the complexity of multiorgan involvement frequently limits treatment response, making extracorporeal membrane oxygenation (ECMO) a critical intervention in highly severe cases.^11^

Although the clinical benefits of ECMO in pediatric respiratory failure are well established, its economic impact in PARDS remains unclear. Existing cost-effectiveness analyses have primarily focused on adult acute respiratory distress syndrome (ARDS), with very limited evidence in pediatric patients and virtually no studies comparing the economic implications of early versus late ECMO initiation.^12,13^ This gap is relevant because the rationale for ECMO, which includes reducing ventilator-induced lung injury and preventing secondary organ dysfunction, suggests potential differences in resource utilization and outcomes depending on the timing of implementation.^14,15^

To address this gap, this study evaluates the cost-effectiveness of early versus late ECMO initiation in pediatric patients with PARDS requiring invasive mechanical ventilation in the intensive care unit (ICU), providing novel evidence to inform clinical decision-making, resource allocation, and economic evaluation in pediatric critical care.

## METHOD

A cost-effectiveness analysis of health technology was conducted to compare early versus late initiation of ECMO in pediatric patients with ARDS admitted to a high-complexity Pediatric Intensive Care Unit (PICU) in northeastern Colombia during 2022. The economic evaluation followed a predefined analytic framework that specified the decision-tree model structure, the cost categories included in the analysis, the effectiveness outcome, and the deterministic and probabilistic sensitivity analyses used to evaluate parameter uncertainty. All patients included in the study required ECMO support as part of their management. Early ECMO was defined as initiation within the first seven days of invasive mechanical ventilation, whereas late ECMO was defined as initiation after seven days. This operational definition was aligned with the clinical objective of assessing whether earlier ECMO deployment resulted in more favorable cost and survival outcomes.

Inclusion criteria included pediatric patients (<18 years) diagnosed with PARDS who met severity thresholds (Phoenix, Pediatric Logistic Organ Dysfunction 2 (PELOD-2), and Murray scores >2.5) and Pediatric Acute Lung Injury Consensus Conference (PALICC) classification >16 and subsequently required ECMO support.^16,17,18^ Patients who died within the first 48 hours after ECMO initiation were excluded to ensure that cost and survival estimates reflected medium-term treatment effects.

Clinical, demographic, and cost data were retrospectively collected and extracted from electronic medical records and hospital billing systems. Data collection followed a standardized verification process to ensure accuracy of clinical variables and completeness of cost entries. The economic evaluation was conducted from the *hospital (provider) perspective*, capturing only direct medical costs incurred during hospitalization. These included ECMO supplies and perfusion services, intensive care and hospital ward stays, laboratory testing, imaging, medications, blood products, and medical fees. Indirect, non-medical, and post-discharge costs were not included because they are not systematically recorded in institutional financial databases. All costs were converted from Colombian pesos (COP) to U.S. dollars using the monthly average exchange rate for 2022.

The statistical analysis included descriptive analyses of categorical and continuous variables, using the chi-square or Mann-Whitney U test according to variable distribution. Comparisons were conducted exclusively between early and late ECMO groups, based on clinically relevant outcomes such as survival at discharge and up to 3 months, ECMO duration, and total hospitalization time. Statistical significance was established at p<0.05. Analyses were performed in STATA version 16. To reduce potential information bias, data coding and consistency were reviewed before analysis, and appropriate statistical tests were selected according to variable type and distribution.

Effectiveness was defined as short-term survival up to 90 days following ECMO initiation. This time horizon was selected because it reflects the maximum follow-up available in this retrospective cohort and is appropriate for a short-term, within-hospital cost-effectiveness framework. Long-term outcomes, including life-years or quality-adjusted life-years (QALYs), were not incorporated because post-discharge functional outcomes, neurodevelopmental trajectories, and validated pediatric utility weights for ECMO survivors were unavailable. Health utility weights were not available for this pediatric population; therefore, QALYs were not estimated. Because the analytic time horizon was limited to 90 days, discounting of costs and outcomes was not applied.

Cost-effectiveness was evaluated using a decision-tree model constructed in Amua v0.3.1, incorporating survival probabilities at discharge and at 30, 60, and 90 days after ECMO initiation. Costs, converted from COP to United States Dollars (USD) using monthly 2022 exchange rates, included components of direct care such as blood bank services, hospital stays, diagnostic tests, medical fees, ultrasounds, medications, and other items. The 2022 Mandatory Traffic Accident Insurance (SOAT) and Injury Severity Score (ISS) rates, adjusted by 30% according to Instituto de Evaluación Tecnológica en Salud (IETS) guidelines, were used to provide a contextualized approximation of costs. The incremental cost-effectiveness ratio (ICER) was calculated by comparing early versus late ECMO initiation. The model assumed that patients required a single ECMO episode and that no additional extracorporeal support runs occurred within the analytic horizon. The analysis assumed that patients did not present with multi-organ dysfunction requiring additional support and that ECMO was implemented by the same specialized team, ensuring extensive expertise in this intervention. A willingness-to-pay threshold equivalent to three times the Colombian Gross Domestic Product (GDP) per capita was applied according to national recommendations from the IETS.

A deterministic sensitivity analysis was conducted, including univariate and bivariate evaluations of key cost and effectiveness parameters, and results were presented in a tornado diagram to illustrate the relative influence of each variable on the ICER. In addition, a probabilistic sensitivity analysis using a Monte Carlo simulation model was performed to assess joint uncertainty in costs and effectiveness for early versus late ECMO initiation, assigning random values to predefined parameter distributions for incremental costs and survival probabilities. Results were summarized in the cost-effectiveness acceptability curve, which illustrates the probability of early ECMO being cost-effective across a range of willingness-to-pay thresholds. Subgroup analyses were not performed due to the limited sample size and the observational design of the study. Distributional or equity effects across population groups were not evaluated. Patients, caregivers, or other stakeholders were not involved in the design or conduct of the economic evaluation.

The study protocol was approved by the Institutional Ethics Committee on October 7, 2024, in accordance with the ethical principles outlined in the Declaration of Helsinki. Data confidentiality was ensured, and all direct identifiers were removed from patient records. The ethics approval number was CEI-2024-08608.

## RESULTS

Seventy-eight patients met the inclusion criteria. Of these, 57.69% were male; 53.85% of patients were covered under the subsidized regime as the main type of insurance in the General System of Social Security in Health; and 94.87% were from Colombia. Most patients (56.41%) were transported by air. Sixty-five patients were included in the early ECMO group (83.33%), and 13 in the late ECMO group (16.67%). However, in the early ECMO group, air transport predominated (64.62%), whereas in the late ECMO group, ground transport prevailed (84.62%), with a statistically significant difference (p=0.001) (Table 1).

**Table 1:** Characteristics of patients with early and late extracorporeal membrane oxygenation.

Variable	Categories	Early ECMO n (%)	Late ECMO n (%)	Total n (%)	p-value
Sex	Female	29 (44.62)	4 (30.77)	33 (42.31)	0.272^†^
	Male	36 (55.38)	9 (69.23)	45 (57.69)	
Age*	Pediatric* (months)	5 (0–16)	2 (0–3)	2.5 (0–3)	0.146^‡^
	Neonatal* (days)	17 (5–48)	6 (6–9.6)	19.5 (6–96)	0.527^‡^
Patient type	Pediatric	58 (89.23)	6 (46.15)	64 (82.05)	<0.001^†^
	Neonatal	7 (10.77)	7 (53.85)	14 (17.95)	
Safety regimen	Contributory	0.28 (43.08)	2 (15.38)	30 (38.46)	0.132^§^
	Subsidized	33 (50.77)	9 (69.23)	42 (53.85)	
	Other	4 (6.15)	2 (15.38)	6 (7.69)	
Country	Foreign	2 (3.08)	2 (15.38)	4 (5.13)	0.066^§^
	Colombia	63 (96.92)	11 (84.62)	74 (94.87)	
Transfer	Air	42 (64.62)	2 (15.38)	44 (56.41)	0.001^§^
	Ground	23 (35.38)	11 (84.62)	34 (43.59)	

ECMO: extracorporeal membrane oxygenation.

*Median (interquartile range);

^†^Test ꭓ^2^;

^‡^Mann-Whitney U test;

^§^ Fisher’s Exact Test.

Source: Prepared by the authors.

Regarding ECMO therapy, the venovenous modality was the most common, observed in 79.49% of cases, with similar distributions between the groups: 76.92% in the early ECMO group and 92.31% in the late ECMO group (p=0.411). As for the indication, ECMO predominated in the early ECMO group (78.46%), whereas cardiovascular ECMO was more frequent in the late group (61.34%), representing a statistically significant difference (p=0.006).

Mortality analysis showed differences between the early and late ECMO groups. Discharge mortality was 33.85% (22 cases) in the early group and 53.85% (seven cases) in the late group, totaling 37.18% (29 cases); however, this difference was not statistically significant (p=0.173). At 30 days, there was one death (1.54%) in the early group and none in the late group (p=0.374). At 2 and 3 months, the survival rates were similar: 63.08% in the early group and 46.15% in the late group, with no significant differences (p=0.255).

In terms of ECMO days, the median was significantly higher in the early group (9 days; interquartile range [IQR]: 6–17) than in the late group (4 days; IQR: 2–6), with a p-value <0.001. The median length of stay in the regular hospital ward was less than 1 day in both groups, with no significant differences (p=0.544). The median hospitalization time was 34 days (IQR: 21–65) in the early ECMO group and 74 days (IQR: 48–103) in the late ECMO group, although this difference was not statistically significant (p=0.087).

Cost analysis comparing early and late ECMO treatments showed no statistically significant differences in most categories evaluated. For blood products, the average costs were 993.59 USD for early ECMO and 1517.16 USD for late ECMO (p=0.449). For ultrasounds, the early group had an expense of 30.51 USD compared with 42.28 USD in the late group (p=0.337). High-cost surgical supplies averaged 5545.84 USD in the early ECMO group and 6039.33 USD in the late group (p=0.208). The costs of hospital stays and medical fees also showed no significant differences, with respective values of 6509.32 USD (early) and 4676.37 USD (late) for hospital stays (p=0.450) and 174.06 USD (early) and 107.93 USD (late) for medical fees (Table 2).

**Table 2:** Cost comparison of early and late extracorporeal membrane oxygenation.

Service	Early ECMO (USD)	Late ECMO (USD)	Total (USD)	p-value
Blood bank	993.59	1517.16	1096.35	0.449
Ultrasounds	30.51	42.28	41.12	0.337
High-cost surgical equipment	5545.84	6039.33	5592.84	0.208
Hospital stay units	6509.32	4676.37	6344.82	0.450
Medical fees	174.06	107.93	172.45	0.529
Clinical laboratory	1460.74	2207.2	1507.15	0.291
Medications	4746.87	4370.88	4605.87	0.489
Oxygen and gases	1180.69	997.57	1171.48	0.397
Perfusion	6438.82	3313.41	5968.83	0.065
Total value	33369.05	43238.77	34074.03	0.777

ECMO: extracorporeal membrane oxygenation; USD: dollars.

Source: Prepared by the authors.

For clinical laboratory expenses, the early ECMO group had a cost of 1460.74 USD compared with 2207.2 USD in the late group (p=0.291). Medication costs were also similar, with 4746.87 USD in the early ECMO group and 4370.88 USD in the late group (p=0.489). Costs associated with oxygen and gases were 1180.69 USD for the early group and 997.57 USD for the late group (p=0.397). Perfusion cost averaged 6438.82 USD in the early group and 3313.41 USD in the late group (p=0.065). The total cost in U.S. dollars was 33,369.05 USD for the early group and 43,238.77 USD for the late group (p=0.777), with no significant difference in either case (Table 2).

**Figure 1:**
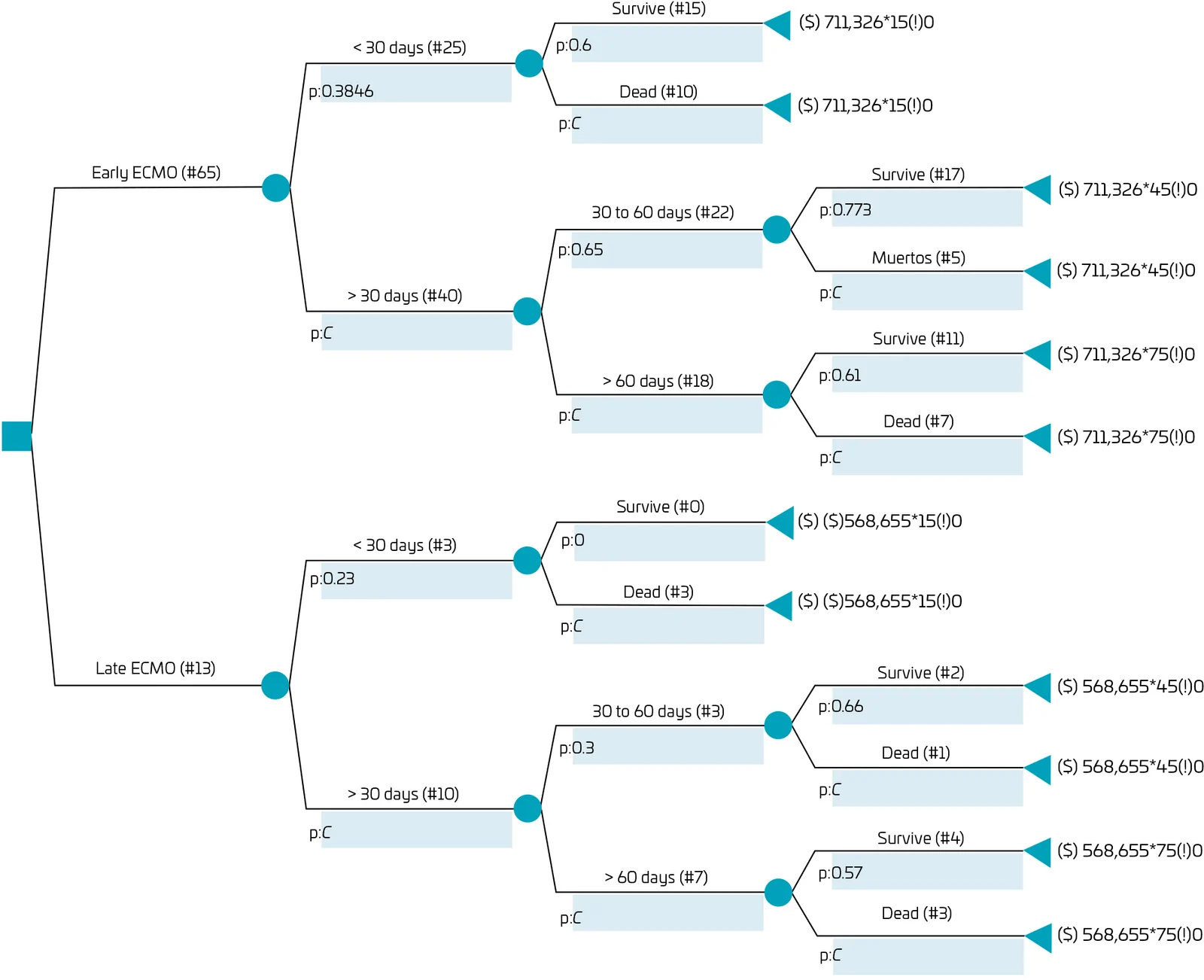
Decision tree model comparing early versus late extracorporeal membrane oxygenation initiation.

The cost-effectiveness of early and late ECMO therapies is presented in Figure 1. Variations in treatment costs had the greatest impact on the economic results, with changes in the estimated cost of early ECMO generating the widest variation in expected value, followed by late ECMO. Adjustments in short-term survival probabilities for both strategies resulted in moderate changes, whereas modifications in other clinical effectiveness assumptions and secondary cost components showed minimal influence on model outputs. These findings indicate that the economic results are predominantly driven by cost-related parameters rather than survival differences within the modeled scenario.

The decision tree illustrates the short-term survival pathways (<30, 30–60, and >60 days) and associated direct medical costs for patients receiving early ECMO (n=65) and late ECMO (n=13). Each terminal node shows the probability of survival or death and the corresponding treatment costs (USD) derived from institutional billing records.

ECMO: extracorporeal membrane oxygenation.

**Source:** Prepared by the authors.

**Figure 2:**
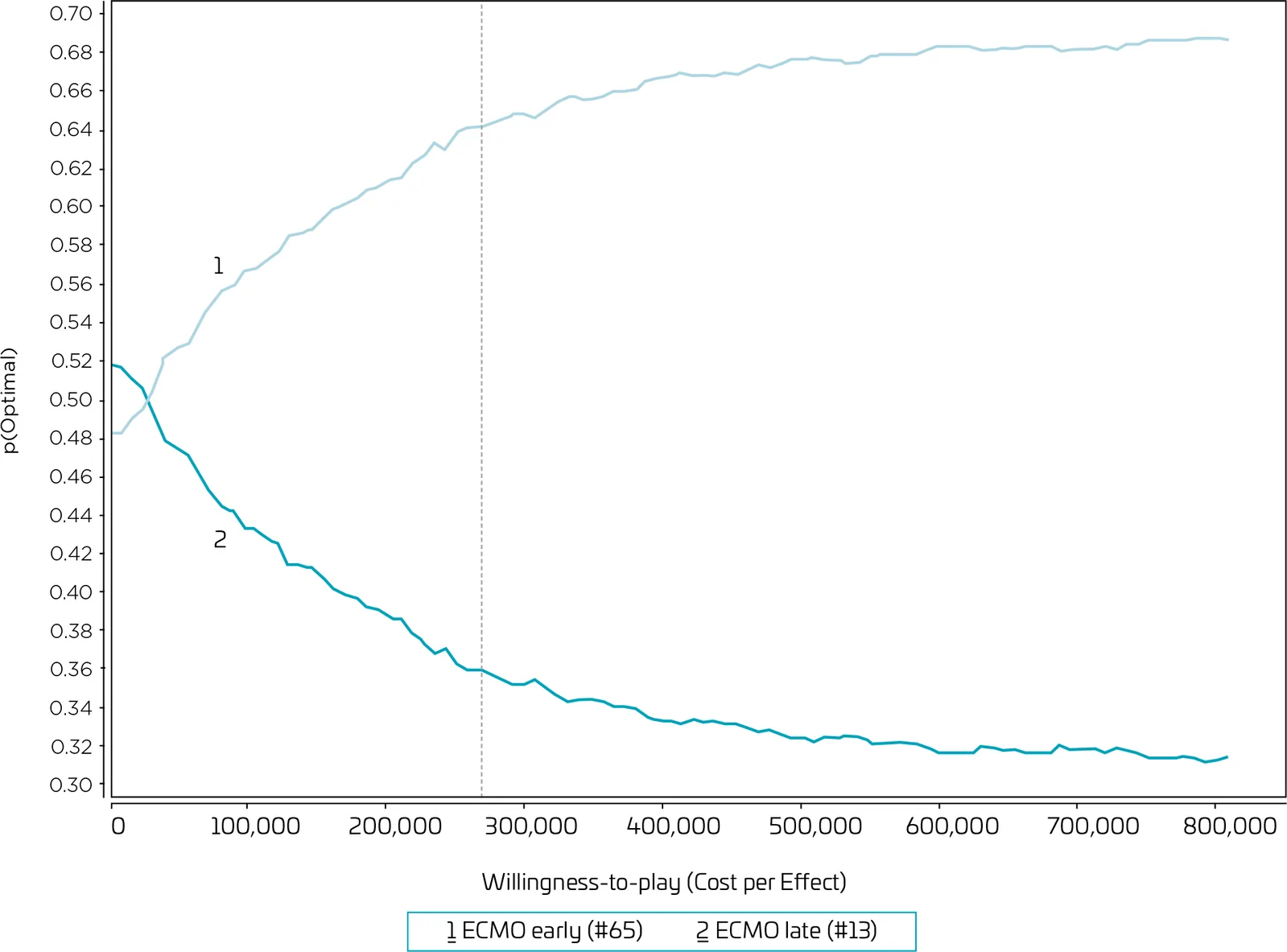
Cost-effectiveness acceptability curve for early versus late extracorporeal membrane oxygenation initiation.

Using the acceptability curve, the probability of achieving an optimal outcome when applying early and late ECMO treatment was determined, showing that early ECMO intervention has a high probability of being cost-effective across willingness-to-pay thresholds (Figure 2).

The Cost-effectiveness acceptability curve displays the probability that early ECMO (curve 1) or late ECMO (curve 2) is the optimal strategy across a range of willingness-to-pay (WTP) thresholds. Each probability is derived from 10,000 Monte Carlo simulations incorporating uncertainty in costs and survival outcomes.

ECMO: extracorporeal membrane oxygenation.

**Source:** Prepared by the authors.

The probabilistic analysis, performed using Monte Carlo simulations, modeled the uncertainty in both costs and effectiveness for early and late-ECMO strategies. The results demonstrated an approximate 21% difference in effectiveness between the interventions, with early ECMO achieving higher clinical effectiveness values. The incremental cost-effect plot positioned early ECMO in the quadrant reflecting both higher cost and greater effectiveness, whereas late ECMO remained close to the origin, indicating lower cost but also substantially lower effectiveness. The relationship between incremental effect and incremental cost showed a clear upward trend, suggesting that increases in effectiveness with early ECMO are consistently associated with higher cost across the simulated scenarios.

## DISCUSSION

ECMO provides temporary pulmonary and/or cardiac support for patients who do not respond to conventional management and has become an essential alternative in critical care settings.^8,11^ In this cost-effectiveness analysis comparing the timing of ECMO initiation, early ECMO demonstrated a trend toward lower costs and greater short-term effectiveness than late ECMO, although differences in mortality and total hospital costs did not reach statistical significance.

Patients who received early ECMO had a markedly shorter median hospital stay (34 vs. 74 days). Although this difference was not statistically significant, the direction of the effect aligns with prior studies indicating that earlier initiation of extracorporeal support may mitigate the progression of organ dysfunction and reduce ICU resource utilization.^19^ The lack of statistical significance in our findings is likely related to the small size of the late ECMO group and notable baseline imbalances, including a higher proportion of neonates and cardiovascular indications among late ECMO patients, factors known to influence severity, trajectory of illness, and overall hospital course.

Survival trajectories also differed between the groups. Early ECMO patients experienced an initial decline in survival during the first 40 days but demonstrated subsequent stabilization over time.^20,21^ This pattern contrasts with the Colombian ECMO consensus, where emergency ECMO following deterioration under conventional management was associated with substantially higher mortality (57 vs. 34%).^22^ Importantly, those national data reflect adult populations and include a larger proportion of high-risk cases requiring urgent cannulation. These contextual differences in age, underlying pathophysiology, and timing of referral likely explain the divergent mortality patterns observed across studies.

From an economic standpoint, the ICER for early ECMO fell below the willingness-to-pay threshold established by the IETS,^23^ indicating a potentially favorable short-term cost-effectiveness profile. However, the absence of statistically significant differences in total hospital costs (33,369 USD vs. 43,238 USD; p=0.777) suggests that these findings should be interpreted cautiously. Cost differences in ECMO are influenced not only by timing of initiation but also by patient complexity, circuit duration, transport logistics, and institutional workflows, all of which varied between groups in this study.^24,25^ Taken together, these results indicate that early and late ECMO were economically comparable within this cohort. At the same time, the consistent directional trend favoring early ECMO may represent a signal that was not statistically detectable because of limited statistical power and the possibility of a type II error.

International economic evaluations demonstrate considerable variability in the cost-effectiveness of ECMO. In Colombia, an analysis of adult patients with ARDS reported an ICER that exceeded national willingness-to-pay thresholds, highlighting the high financial burden of this therapy in resource-constrained settings.^3^ Likewise, international studies reveal substantial heterogeneity in cost per QALY: a prospective evaluation from Germany showed that adult ECMO survivors incurred mean first-year costs exceeding USD 200,000, largely driven by intensive care requirements and post-hospital rehabilitation,^24^ while a Taiwanese study of 919 ECMO-treated adults demonstrated wide variation in cost-effectiveness across risk groups, with cost per QALY ranging from USD 24,230 in lower-risk patients to over USD 149,000 among those with high predicted mortality.^26^ Together, these findings underscore that ECMO’s economic value is highly context-dependent and shaped by patient selection, institutional expertise, and healthcare system financing. Within this broader evidence landscape, our study contributes early pediatric data from a middle-income setting, suggesting that early ECMO may offer short-term economic advantages — though confirmation through larger, better-adjusted analyses remains essential.

An additional consideration when interpreting these findings is that ECMO is fundamentally a complex life-support technology whose indication is not determined solely by standardized criteria but also involves a degree of clinical judgment. Decisions regarding ECMO initiation are highly influenced by the experience and expertise of the multidisciplinary healthcare team, institutional infrastructure, availability of specialized resources, and patient-specific clinical characteristics. These contextual factors can substantially shape both outcomes and resource utilization and may be decisive regardless of formal cost-effectiveness estimates. Therefore, economic evaluations of ECMO should be interpreted within the broader framework of institutional capacity and real-world clinical decision-making environments, particularly in middle-income settings where variability in access and expertise may significantly affect the applicability of cost-effectiveness results.

This study has some limitations. The two groups differed substantially in baseline characteristics, including age distribution, ECMO indication, and transport modality, introducing unadjusted confounding and limiting causal inference. The late ECMO group was small (n=13), reducing statistical power and increasing the risk of type II error. In addition, this short-term, hospital-based evaluation did not incorporate post-discharge survival, neurodevelopmental outcomes, readmissions, indirect costs, or health-related quality of life measures, precluding the calculation of long-term survival or QALYs. The limited availability of validated pediatric utility weights further restricts QALY-based extrapolations in this population.

Nevertheless, these findings contribute preliminary, real-world evidence from a middle-income setting, addressing a gap in economic evaluations related to the timing of ECMO initiation in PARDS. Further research should incorporate prospective multicenter cohorts, group-balancing methods, long-term follow-up, and economic modeling that includes downstream costs and functional outcomes to inform clinical and policy decision-making regarding ECMO resource allocation in high-acuity pediatric care.

In conclusion, early and late ECMO initiation were associated with comparable short-term survival and in-hospital costs in pediatric patients with PARDS. Although a directional trend favoring early ECMO was observed in economic modeling, this pattern did not reach statistical significance and may reflect limited statistical power. These findings suggest that a true difference between strategies cannot be excluded and warrants investigation in larger, adequately powered studies.

## Data Availability

The data that support the findings of this study are available from the corresponding author upon reasonable request. The datasets are not publicly available due to institutional policies regarding patient confidentiality.
